# TELAPSI: Development and Content Validation of a Digital Learning Object Based on Cinematic Narratives for Schizophrenia Education in Medicine

**DOI:** 10.1007/s40670-026-02915-0

**Published:** 2026-10-11

**Authors:** José Maria Santiago da Silva Júnior, João Wallace Carvalho de Oliveira, Raquel Nunes de Alencar, Ana Thais Lima Farias, Marina Marques Maia, Gustavo Coelho Peixoto, Marcos Kubrusly, Arnaldo Aires Peixoto Junior

**Affiliations:** 1 School of Medicine, Christus University, Fortaleza, Ceará Brazil; 2 Department of Clinical Medicine, School of Medicine, Federal University of Ceará, Prof. Costa Mendes Street, 1608 - 4th floor - Rodolfo Teófilo, Fortaleza, 60430-140 CE Brazil

**Keywords:** Medical education, Schizophrenia, Motion pictures, Educational technology, Psychopathology, Learning

## Abstract

**Objective:**

To develop and evaluate TELAPSI, a freely accessible bilingual digital learning object that supports faculty in teaching about schizophrenia through curated cinematic scenes linked to psychopathological descriptions and structured lesson plans.

**Methods:**

A methodological study with a quantitative and qualitative approach was conducted in three sequential phases: intentional film selection, platform development, and evaluation by a panel of 20 experts (10 psychiatrists and 10 health educators). Content was assessed with the Brazilian-validated Learning Object Review Instrument (LORI) and usability with the System Usability Scale (SUS); groups were compared using the Mann–Whitney test and Welch’s t-test, and open-ended responses were examined through descriptive qualitative analysis.

**Results:**

Median LORI domain scores ranged from 4.40 to 4.90 (1–5 scale), with content quality, motivation, and usability and interaction rated highest. Significant differences between groups appeared only in usability and interaction (*p* = 0.034) and accessibility (*p* = 0.009), with health educators scoring higher than psychiatrists. The mean SUS score was 90.4 (95% confidence interval [CI] 86.1–94.7), classified as excellent, again higher among health educators (95.5 vs. 85.3; *p* = 0.010; Cohen’s d = 1.33). Qualitative analysis identified three themes: usability and user experience; pedagogical relevance and innovation; and suggestions for improvement and expansion.

**Conclusion:**

TELAPSI achieved high expert-rated quality as a digital learning object and excellent usability, providing a transferable model for film-based teaching in psychiatric education. These findings establish its content validity and usability as the validation phase preceding controlled studies of learner outcomes.

**Supplementary Information:**

The online version contains supplementary material available at https://doi.org/10.1007/s40670-026-02915-0.

## Introduction

The teaching of psychiatry in undergraduate medical education has long confronted structural limitations related to the nature of its primary objects of study. Unlike other medical specialties, psychiatry demands the recognition and interpretation of subjective phenomena, including perceptual disturbances, thought disorganization, affective blunting, and identity alterations, that are not easily transmitted through conventional didactic methods [1]. Lectures, textbooks, and brief clinical clerkships, though indispensable, frequently fall short in conveying the phenomenological depth of psychiatric illness, especially among learners with limited exposure to clinical environments [2].

Schizophrenia exemplifies these pedagogical difficulties with acuity. Characterized by a heterogeneous constellation of positive, negative, disorganized, and cognitive symptoms, the disorder presents the educator with a unique challenge: how to convey experiences that are, by definition, profoundly alien to most learners’ lived realities [3]. Conceptual descriptions of delusions or auditory hallucinations may be intellectually acquired without generating genuine clinical understanding or empathic resonance. Furthermore, students frequently arrive with preconceived, often stigma-laden images of schizophrenia derived from popular culture [4].

Cinematic narratives have emerged as a compelling pedagogical resource to address these challenges. Films afford the viewer sustained exposure to psychiatric phenomena as they unfold across time, through facial expressions, behavioral patterns, interpersonal dynamics, and narrative context, elements that collectively produce a form of experiential understanding difficult to achieve through written description alone [5, 6].

Nevertheless, the existing literature reveals persistent gaps in the operationalization of cinema-based pedagogy. Most reported initiatives are isolated and methodologically unsystematic, relying on ad hoc film selection and unstructured discussion [7]. To our knowledge, digital platforms integrating psychopathological analysis, clinical correlation, and guided pedagogical frameworks within a single tool for psychiatry instruction remain scarce. This lacuna appears particularly critical for schizophrenia, a condition whose teaching would benefit most from visual, narrative, and phenomenologically grounded approaches.

The present study reports the development and evaluation of TELAPSI, a digital educational platform conceived to support the teaching about schizophrenia through curated cinematographic scenes. The platform integrates structured lesson plans, didactic guides, psychopathological descriptions, and clinical correlations, offering faculty a systematized and theoretically grounded tool for incorporating cinema into psychiatric education.

## Materials and Methods

### Study Design

This methodological study employed a quantitative and qualitative approach to develop and evaluate a digital educational tool designed for teaching about schizophrenia. The study was organized into three sequential phases: (1) mapping and intentional sampling of films based on thematic relevance, clinical accuracy, and pedagogical potential; (2) development of a structured digital educational tool; and (3) pedagogical, technological, user experience and usability evaluation by experts.

### Film Selection Criteria

In this study, cinematic narratives refer to particular film scenes, rather than complete films, selected for their capacity to portray specific psychopathological phenomena with clinical accuracy. Film selection followed intentional, criterion-based sampling: candidates were evaluated for thematic relevance to schizophrenia phenomenology, accuracy of symptom portrayal, and pedagogical potential for mapping to defined learning objectives. Cultural popularity and narrative appeal alone were not treated as sufficient selection criteria; priority was given to clinical accuracy, psychopathological richness, and anti-stigma framing over box-office salience, consistent with structured curation frameworks described in the literature [8]. Selected scenes were catalogued with defined timestamps and linked to specific competencies, allowing faculty to deploy individual segments aligned with session objectives rather than requiring full-film screening.

### Platform Development: TELAPSI

TELAPSI (an acronym combining Tela/Screen and Psi/Psychiatry) was conceived as a digital support platform for faculty teaching about schizophrenia through cinema. The platform was structured around four interconnected components: (1) a curated catalogue of cinematographic scenes with detailed psychopathological description and clinical commentary; (2) didactic guides organized by learning objective, student level, and session duration; (3) structured lesson plans adaptable to face-to-face, hybrid, and remote teaching modalities; and (4) pedagogical orientations linking scenes to clinical competencies.

### Participants

A purposive expert panel of 20 specialists (10 psychiatrists and 10 health educators) was convened for platform evaluation. Panel members were recruited through snowball referral among faculty, beginning with two randomly selected seed contacts: one via the Brazilian Association of Psychiatry’s mailing list (psychiatrist) and the other via the University Master’s program in health education (health educator). Each seed contact then referred colleagues who met the eligibility criteria until ten participants per group were enrolled. Selection criteria for psychiatrists included: specialist certification by the Brazilian Association of Psychiatry or completion of residency training at least five years prior, plus documented teaching experience. Selection criteria for health educators included: minimum master’s degree in education, health education, or related fields, with demonstrated experience in active methodologies or educational technologies.

### Instruments

The pedagogical, technological, and user experience aspects of the platform were assessed using the Brazilian-validated version of the Learning Object Review Instrument (LORI) [9], comprising eight dimensions (content quality, learning objective alignment, feedback & adaptation, motivation, presentation design, usability & interaction, accessibility, and standards compliance), each rated on a 5-point Likert scale. Usability was assessed using the System Usability Scale (SUS) [10, 11], a 10-item validated instrument yielding scores from 0 to 100. A score of 68 represents an acceptable threshold, while scores above 85 are classified as excellent. This scale was selected over alternative usability metrics for its technology-agnostic design, extensive cross-domain validation, brevity, which limits respondent burden when paired with the eight-dimension LORI in a single evaluation protocol, and standardized benchmarking thresholds that support direct interpretation of scores [10, 11]. Both instruments were administered electronically.

### Ethical Aspects

The study was approved by the Research Ethics Committee of Universidade Christus (CAAE: 80434224.6.0000.5049). All participants provided written informed consent prior to evaluation.

### Data Analysis

Data distribution was evaluated using the Shapiro–Wilk test. Continuous variables with normal distribution were described with mean and standard deviation (SD), while non-normally distributed variables were summarized as median and interquartile range (IQR). Quantitative analysis of LORI scores included descriptive statistics (median and IQR). Intergroup comparisons between psychiatrists and health educators were performed using the Mann-Whitney test. SUS scores were analyzed descriptively (mean, SD and confidence intervals), and between-group comparison was conducted using the Welch t-test and confirmed with Mann-Whitney. Effect size was estimated using Cohen’s d. Internal consistency was assessed via Cronbach’s alpha. Qualitative analysis of open-ended responses employed Descriptive Qualitative Analysis [12], following iterative cycles of data familiarization, meaning unit identification, thematic grouping, and descriptive synthesis.

### Use of Artificial Intelligence

During the preparation of this manuscript, the authors used artificial intelligence-assisted technologies solely to improve the readability and language of the text. These tools complemented, and did not replace, the authors’ writing, and were not used to generate or analyse study data or to draw scientific conclusions. The authors reviewed and edited the resulting text and take full responsibility for the content of the publication.

## Results

### Platform Description

The TELAPSI platform was developed as a structured digital environment to support teaching about schizophrenia through film. In its final version, the platform consists of modules that include teaching guides with scene-by-scene psychopathological analysis and clinical correlation; lesson plans organized by content area, educational level, and session format; and guidance materials linking the cinematic content to competency frameworks. The included films represent various phenomenological dimensions of schizophrenia, allowing for selective use according to educational objectives. The platform was designed for access via desktop browsers and mobile devices, without the need for institutional credentials, in a bilingual format (English and Portuguese) and is available at https://telapsi-lit-2025.web.app/.

### LORI Content Validation

All eight domains received high scores, with the median ranging from 4.40 to 4.90 on the 1–5 scale. Content quality, motivation and usability & interaction attained the highest scores. The lowest scores, though still high in absolute terms, were observed for feedback & adaptation and accessibility. Stratified analysis by evaluator group revealed generally concordant assessments between health educators and psychiatrists across most domains. The sole statistically significant intergroup difference was observed in the usability & interaction (*p* = 0.034), and accessibility domain (*p* = 0.009), with health educators assigning higher scores than psychiatrists. No significant differences were detected in any other domain (all *p* > 0.05). Full results are presented in Table 1.

**Table 1:** Learning object review instrument (LORI) domain scores by evaluator group (*N* = 20)

Domain	All the experts (*N* = 20)	Health educators (*N* = 10)	Psychiatrists (*N* = 10)	*p* value
Content quality	5.00 (5.00–5.00)	5.00 (5.00–5.00)	5.00 (5.00–5.00)	0.167
LO alignment	5.00 (4.75–5.00)	5.00 (5.00–5.00)	5.00 (4.00–5.00)	0.144
Feedback & adaptation	4.50 (4.00–5.00)	4.50 (4.00–5.00)	4.50 (4.00–5.00)	1.000
Motivation	5.00 (5.00–5.00)	5.00 (5.00–5.00)	5.00 (5.00–5.00)	1.000
Presentation design	5.00 (4.00–5.00)	5.00 (5.00–5.00)	5.00 (4.00–5.00)	0.366
Usability & interaction	5.00 (5.00–5.00)	5.00 (5.00–5.00)	5.00 (4.00–5.00)	0.034*
Accessibility	4.50 (4.00–5.00)	5.00 (5.00–5.00)	4.00 (4.00–4.00)	0.009*
Standards compliance	5.00 (4.00–5.00)	5.00 (5.00–5.00)	5.00 (4.00–5.00)	0.366

Note: Data presented as median (IQR); Mann-Whitney test (Health educators vs. Psychiatrists); * *p* < 0.05

### Usability Assessment

The mean SUS score was 90.4 (SD = 9.2; 95% CI: 86.1–94.7), indicating excellent usability according to established benchmarks [11, 13]. The Cronbach’s alpha coefficient for the score was 0.87, reflecting high internal consistency. Health educators assigned significantly higher usability scores than psychiatrists (95.5 ± 5.1 vs. 85.3 ± 9.6; Welch t-test: *p* = 0.010; Mann-Whitney: *p* = 0.018), with a large effect size (Cohen’s d = 1.33), supported by a strong rank-biserial correlation (*r* = 0.63), reinforcing the robustness of the observed effect across parametric and nonparametric approaches. Despite this difference, both groups scored well above the 68-point adequacy threshold and the 85-point excellence threshold. Results are presented in Table 2.

**Table 2:** System usability scale (SUS) scores by evaluator group

Group	Score	*p* value
Health educators (*N* = 10)	95.5 (5.1)	—
Psychiatrists (*N* = 10)	85.3 (9.6)	0.010*

Note: Data presented as mean (SD), Welch t-test, confirmed by Mann-Whitney (*p* = 0.018); Cohen’s d = 1.33; * *p* < 0.05

### Qualitative Analysis

Eighteen valid open-ended responses were analyzed using Descriptive Qualitative Analysis, generating three primary themes. The first theme, usability and user experience, was characterized by predominantly positive assessments. Evaluators described the platform as simple, intuitive, and visually organized. Minor concerns were noted regarding navigation on mobile devices and minor textual revision needs. The second theme, pedagogical relevance and innovation, reflected broad consensus regarding the platform’s educational value. Evaluators recognized TELAPSI as an innovative tool that effectively bridges audiovisual content and clinical knowledge, facilitating active, contextually grounded learning. The third theme, suggestions for improvement and expansion, encompassed recommendations for enhancing interactivity (e.g., comment sections and peer discussion forums), enriching assessment resources, and expanding scope beyond schizophrenia. The possibility of embedding audiovisual content directly into the platform was raised, alongside acknowledgment of copyright constraints.

## Discussion

The present study demonstrates that TELAPSI achieved high scores in its evaluation as a learning object (LO) and received excellent usability ratings in its initial assessment by experts. These findings position TELAPSI as a methodologically grounded contribution to cinema-based psychiatric education and extend the range of digital educational technologies for use in training of health professions.

The domains yielding a significant intergroup difference were accessibility and usability & interaction, with health educators assigning higher ratings than psychiatrists. This finding may reflect differential familiarity with digital educational platforms: educators in health professions programs routinely engage with Learning Management Systems. Psychiatrists may apply implicitly higher standards derived from clinical software environments characterized by greater robustness. The somewhat wider confidence interval in the psychiatry subgroup for accessibility suggests greater variability in perceptions among clinical evaluators.

The relatively lower scores in feedback & adaptation and accessibility signal areas for iterative development. Qualitative findings contextualize these results: evaluators specifically identified the absence of embedded assessment instruments, the lack of learner progress tracking, and restricted mobile navigation as gaps warranting attention. These concerns are consistent with broader evidence on digital LO quality [14], which consistently identifies feedback mechanisms and adaptive functionality as the most demanding dimensions for educational tool developers to implement. Future iterations of TELAPSI should incorporate learning outcome measurement tools and adaptive content sequencing.

The SUS mean score classifies TELAPSI as exhibiting excellent usability [11, 13]. This result is significant given that the evaluating panel included psychiatrists with potentially limited prior experience with specialized educational platforms. The Cronbach’s alpha confirms robust internal consistency of usability ratings. The significant difference in these scores between health educators and psychiatrists merits careful interpretation. While both groups exceeded the excellent threshold, the large effect size indicates a meaningful practical difference. These findings suggest that future versions of TELAPSI could benefit from usability testing to identify platform features that better meet the specific interaction preferences of psychiatrists interested in using this resource to teach trainees about schizophrenia.

Qualitative findings reinforced the quantitative results. The identification of pedagogical relevance and innovation as a recurrent theme signals that evaluators recognized TELAPSI’s core educational proposition, the structured integration of cinematographic narrative with psychopathological analysis, as distinctive and valuable. This perception aligns with Calzada et al. [15], who demonstrated that mainstream films used in structured psychiatric seminars produced superior outcomes compared to unstructured exposure, particularly regarding motivation and clinical nuance recognition. The expressed desire for assessment instruments is particularly significant from an evidence generation perspective: the incorporation of pre/post knowledge tests and clinical reasoning rubrics would transform TELAPSI into a platform capable of producing evaluable learning outcomes aligned with competency-based medical education frameworks [2].

### Film Curation

The intentional curation of films for psychiatric education demands empirically grounded selection criteria, given that the majority of commercially produced cinema misrepresents schizophrenia. Content analysis of English-language films (1990–2010) found that 83% of characters with schizophrenia displayed violent behavior, homicidal conduct appeared in nearly one-third, and positive symptoms were overrepresented while negative symptoms were largely absent [16]. Convergent findings from Bollywood productions confirmed that schizophrenia was the most frequent diagnosis depicted, aggression the dominant symptom, and deterioration the prevailing outcome, patterns that reinforce public misconceptions about psychotic disorders [17]. Cultural popularity and narrative appeal are therefore insufficient selection criteria. Wilson et al. [8] showed that a structured process drawing from 503 candidate films, applying standardized entertainment ratings and psychiatric content mapping against the Global Burden of Disease framework, yields a pedagogically defensible film set, with documentary and biographical formats best suited for conveying phenomenological authenticity without stigmatizing distortion. TELAPSI’s curatorial approach directly reflects this evidence, prioritizing clinical accuracy, psychopathological richness, and anti-stigma framing over cultural salience.

### Scene and Time Counting

Precise scene identification distinguishes structured cinema-based teaching from unmediated film viewing. Blasco et al. [18] formalized this through the curation of individual clips with defined timestamps, enabling targeted exposure to clinically relevant moments without full-film screening. The approach concentrates affective engagement, accommodates session time constraints, allows thematic rather than narrative sequencing, and supports annotated commentary prompts that shift viewing from passive to reflective. TELAPSI operationalizes this principle by cataloguing scenes according to psychopathological content, learning objective, and session duration, allowing faculty to deploy specific film segments aligned with defined curricular goals.

### Advances in Psychiatric Knowledge

Empirical evidence from structured cinemeducation programs consistently demonstrates measurable gains in students’ psychiatric knowledge, diagnostic confidence, and engagement when film is integrated as an active pedagogical tool rather than passive illustration. D’Agostini et al. [19] evaluated a film-based method in a fifth-year psychiatry course at the University of Milan, in which student groups analyzed film sequences mapped to DSM-5 diagnoses. Post-intervention, 97.87% of students reported improved teaching effectiveness and 100% reported enhanced topic comprehension; final examination scores improved significantly in the cohort using the method. Students attributed this gain to film’s capacity to give concrete contours to abstract psychopathological concepts and to support memorization through narrative, consistent with constructivist learning principles. Calzada et al. [15] implemented a ten-session course at the University of Geneva in which students applied DSM-5 criteria to film characters; self-perceived diagnostic confidence improved significantly across sessions, with symptom portrayal richness and empathic identification as independent predictors of learning gain. This finding directly validates TELAPSI’s curatorial framework, in which films were selected on psychopathological richness and clinical precision rather than cultural visibility, and scenes are catalogued by diagnostic content to maximize instructional yield.

### Stigma Reduction

The potential of cinematographic interventions to reduce stigma toward people with schizophrenia represents one of the most clinically significant arguments for their integration into psychiatric education. A single documentary film following individuals with schizophrenia in daily life significantly reduced stereotypes of dangerousness and unpredictability and decreased desired social distance exclusively among viewers, with effects specific to schizophrenia [20]. Importantly, stigma reduction is not an automatic consequence of film exposure: Ritterfeld and Jin [21] showed that an accurate fictional portrayal alone increased knowledge but did not reduce stigma, attitudinal change required the addition of a structured educational supplement delivered after viewing. Conversely, a film focused solely on acute psychotic experience, without contextualizing recovery, paradoxically heightened negative stereotypes and social distancing in some viewers [22]. These findings collectively confirm that the mediating structure surrounding film exposure, not the film itself, determines whether cinematic content produces attitudinal change. TELAPSI’s psychopathological annotations and pedagogical guides operationalize precisely this principle, positioning the platform as a candidate anti-stigma tool. Future implementation studies should incorporate validated stigma measurement instruments to generate quantitative evidence of impact.

### Study Limitations

Several study limitations should be acknowledged. First, the cross-sectional expert panel design does not permit assessment of TELAPSI’s impact on actual student learning or attitudinal outcomes. Future randomized or quasi-experimental studies comparing TELAPSI-augmented instruction with conventional teaching approaches are necessary to establish pedagogical efficacy. Second, the sample of 20 evaluators, while meeting methodological ideal (statistical precision and calculation of robust confidence intervals) for this design according to the literature [10], limits the generalizability of intergroup comparisons. Third, the study was conducted in a single institutional context; cross-cultural validation in other settings is warranted. Fourth, the absence of engagement analytics within the current platform version precludes fine-grained analysis of user behavior.

### Innovation in Psychiatric Education

Despite these limitations, TELAPSI occupies a distinctive position within the existing landscape of cinema-based psychiatric education resources. Unlike prior initiatives that either catalogued films for self-directed student viewing or proposed ad hoc faculty-driven screening sessions, TELAPSI constitutes a formally structured LO, an integrated digital environment that combines curated psychopathological scene analysis, competency-mapped lesson plans, and faculty orientation materials within a single, freely accessible platform. This architecture moves beyond the fragmented and opportunistic use of cinema that characterizes most reported initiatives in the literature, providing a replicable and scalable curricular infrastructure that faculty can adopt without prior expertise in cinemeducation.

## Conclusions

This study developed a digital platform designed to support faculty in teaching about schizophrenia through selected cinematic narratives. The platform, named TELAPSI, received a positive evaluation as a LO and was rated as having excellent usability by experts. TELAPSI fills a gap in the landscape of educational technologies for teaching psychiatry by providing systematized and operationally accessible resources for film-based teaching. Future research should evaluate the platform’s effectiveness in controlled educational settings, examining its impact on the acquisition of psychopathological knowledge, the development of clinical reasoning, the reduction of stigma, and communicative competence. Expansion to other psychiatric conditions, the incorporation of interactive assessment modules, and the development of interfaces optimized for mobile devices represent trajectories for improving the platform.

## Supplementary Information

Below is the link to the electronic supplementary material.

Supplementary Material 1

## Data Availability

The data and materials that support the findings of this study are openly available in the Open Science Framework (OSF) repository at https://osf.io/zskfv/overview? view_only=a7ebcb3289914b699f99c5299035880b.
