# Supplementary material for: TELAPSI: Development and Content Validation of a Digital Learning Object Based on Cinematic Narratives for Schizophrenia Education in Medicine
Source: Med Sci Educ. 2026 Oct 11;36(6):2757–63. doi: 10.1007/s40670-026-02915-0 (PMC13759998; doi:10.1007/s40670-026-02915-0)
Supplement: Supplementary file 1 [file 40670_2026_2915_MOESM1_ESM.docx]

**Response to Reviewers**

Article: "TELAPSI: Development and Content Validation of a Digital Learning Object Based on Cinematic Narratives for Schizophrenia Education in Medicine"

Dear Dr. Wijnen-Meijer,

We thank you and reviewers for the careful evaluation of our manuscript and for the recommendation of acceptance pending minor revisions. We have addressed every point raised below. All wording added or altered in the manuscript in response to these comments is marked in red.

**REVIEWER #1**

*“Thanks for the chance to read your interesting manuscript, and for your attention to themes of stigma related to topics in mental health.”*

**Response:** We appreciate this observation.

**Introduction / background / rationale**

*“To make your manuscript more accessible, I recommend limiting use of abbreviations like SUS and LORI.”*

**Response:** We appreciate this observation. Six further repetitions of LORI and SUS were removed elsewhere in the manuscript or replaced with an unambiguous referring expression.

“Internal consistency ~~of the SUS~~ was assessed via Cronbach's alpha.“ (page 5)

“All eight ~~LORI~~ domains received high scores, with the median ranging from 4.40 to 4.90 on the 1–5 scale. Content” (page 6)

“**~~LORI~~ Domain**” (page 6)

“The Cronbach's alpha coefficient for the ~~SUS~~ score was 0.87, reflecting high” (page 7)

“**~~SUS~~ score**” (page 7)

“significant difference in these ~~SUS~~ scores between health educators and psychiatrists merits careful interpretation.” (page 8)

*“A copyediting thing to watch for throughout - the platform you've developed teaches ABOUT schizophrenia. It isn't "teaching schizophrenia." This is a convention of preposition use in English. Two places to update include line 16, line 39 on page 4.”*

**Response:** Thank you for reading this through carefully and for your instructions. We have made the corrections.

**Feedback on the design, methods and statistics**

*“The methods section would benefit from a paragraph on film selection - some of the content on p. 8 in Discussion would be helpful to see earlier in the manuscript. It wasn't clear to me until then what precisely you meant by "cinematic narratives".*

**Response:** We added a new Methods subsection, “Film Selection Criteria,” immediately after “Study Design” and before “Platform Development”. It defines cinematic narratives operationally and states the selection criteria (thematic relevance, clinical accuracy of symptom portrayal, pedagogical potential).

*How did you recruit participants?*

**Response:** We detail the method used to recruit participants (panel members).

*p. 5, line 21: described WITH mean and SD”*

**Response:** Corrected. “Continuous variables with normal distribution were described as mean and standard deviation” now reads “were described with mean and standard deviation (SD).”

**REVIEWER #2**

**Feedback on the introduction/background/rationale/theoretical framework:**

*“Background regarding representations and misrepresentations of stigmatized mental disorders such as schizophrenia are discussed. While fully lucid initially, by the penultimate paragraph the authors make claims that "guided pedagogical frameworks" remain scarce without a direct supporting citation or depth of explanation that would make such citation unnecessary.”*

**Response:** Thank you for your careful reading. We clarified in the text that the information is based on the authors’ own knowledge and is not a conclusion drawn from the review cited in the previous sentence.

**Feedback on the design, methods and statistics:**

*“A straight-forward, mixed methods approach with parity between health educators and psychiatrists was used to evaluate the authors' proprietary digital learning object. Given the overwhelming number of Likert scale surveys in circulation, the authors would benefit from explaining why SUS was used rather than another metric. However, the directness of the approach is methodologically sound.”*

**Response:** Addressed. We added a justification sentence to the Instruments section. We also took the opportunity to correct a minor subject–verb agreement error identified during this revision (Instruments section: “*aspects of the platform were assessed*”, previously “*was performed*”).

**Feedback on the results/discussion or main body of the manuscript:**

*“Appropriate statistics were used for the reported outcome data. The authors were also thoughtful in interpreting these findings, and cautious enough to not overstate their findings through a concise but fair Limitations section.”*

**Response:** We appreciate your favorable assessment and your comments.

**Feedback on the conclusions/summary:**

*“Conclusions briefly summarize the findings in a fair manner.”*

**Response:** We appreciate your favorable assessment and your comments.

**Feedback on the appropriateness and timeliness of the reference list:**

*“Appropriate and timely for the topic.”*

**Response:** We appreciate your favorable assessment and your comments.

**Feedback on the tables and figures:**

*“Findings were presented very clearly with relevant significance measurements in the Tables.”*

**Response:** We appreciate your favorable assessment and your comments.
